# Augmented reality in the operating room: a clinical feasibility study

**DOI:** 10.1186/s12891-021-04339-w

**Published:** 2021-05-18

**Authors:** Cyrill Dennler, David E. Bauer, Anne-Gita Scheibler, José Spirig, Tobias Götschi, Philipp Fürnstahl, Mazda Farshad

**Affiliations:** 1 Spine Division, University Hospital Balgrist, University of Zürich, Forchstrasse 340, 8008 Zurich, Switzerland; 2 Laboratory for biomechanics, University Hospital Balgrist, University of Zürich, Forchstrasse 340, Zurich, 8008 Switzerland; 3 Computer Assisted Research and Development Group, University Hospital Balgrist, University of Zürich, Forchstrasse 340, Zurich, 8008 Switzerland

**Keywords:** Augmented reality, Navigation, Orthopedics, Osteotomy, Hololens

## Abstract

**Background:**

Augmented Reality (AR) is a rapidly emerging technology finding growing acceptance and application in different fields of surgery. Various studies have been performed evaluating the precision and accuracy of AR guided navigation. This study investigates the feasibility of a commercially available AR head mounted device during orthopedic surgery.

**Methods:**

Thirteen orthopedic surgeons from a Swiss university clinic performed 25 orthopedic surgical procedures wearing a holographic AR headset (HoloLens, Microsoft, Redmond, WA, USA) providing complementary three-dimensional, patient specific anatomic information. The surgeon’s experience of using the device during surgery was recorded using a standardized 58-item questionnaire grading different aspects on a 100-point scale with anchor statements.

**Results:**

Surgeons were generally satisfied with image quality (85 ± 17 points) and accuracy of the virtual objects (84 ± 19 point). Wearing the AR device was rated as fairly comfortable (79 ± 13 points). Functionality of voice commands (68 ± 20 points) and gestures (66 ± 20 points) provided less favorable results. The greatest potential in the use of the AR device was found for surgical correction of deformities (87 ± 15 points). Overall, surgeons were satisfied with the application of this novel technology (78 ± 20 points) and future access to it was demanded (75 ± 22 points).

**Conclusion:**

AR is a rapidly evolving technology with large potential in different surgical settings, offering the opportunity to provide a compact, low cost alternative requiring a minimum of infrastructure compared to conventional navigation systems. While surgeons where generally satisfied with image quality of the here tested head mounted AR device, some technical and ergonomic shortcomings were pointed out. This study serves as a proof of concept for the use of an AR head mounted device in a real-world sterile setting in orthopedic surgery.

**Supplementary Information:**

The online version contains supplementary material available at 10.1186/s12891-021-04339-w.

## Background

Precise positioning of mechanical elements including screws, surgical guides, prosthetic components and anchors to restore anatomy and function remains a desirable goal in orthopedic surgery [1]. Image-based intraoperative techniques such as two-dimensional and three-dimensional (3D) fluoroscopy or Computed tomography (CT) -based navigation increase the precision but also radiation exposure to the patient and operating room personnel [2–8]. Other promising methods of intraoperative navigation techniques including mechanical drilling aids or CAD-designed and 3D-printed patient-specific instruments are cost intense and may require prolonged preoperative preparation and planning [9–11].

**Fig. 1:**
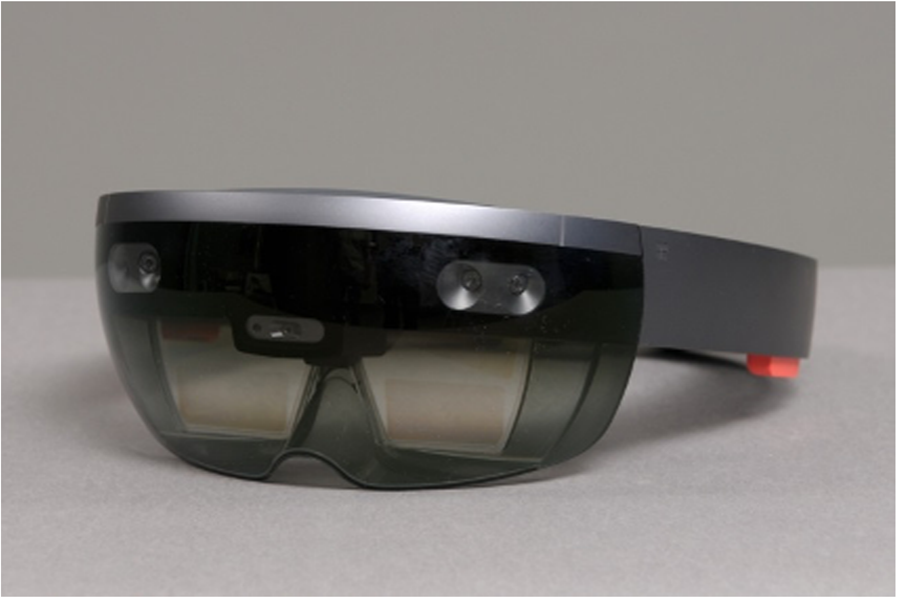
Augmented reality headset (Hololens I, Microsoft, Redmond, WA, USA)

Augmented Reality (AR) is a rapidly emerging technology providing the user with computer-generated information superimposed to real-world environment. The user’s field of view is transformed into a display where real-world objects can be complemented with virtual data. This way information including drilling axis or cutting planes can be projected directly onto the patients’ anatomy within the surgical field. Although its application in orthopedic surgery today remains limited, AR was gradually introduced in different experimental medical and surgical settings [12–16]. Recent studies demonstrated that AR may improve accuracy, safety and efficacy of surgical procedures [17, 18]. In order to ultimately provide a benefit for the patient this novel technology is required to support the surgeon in his decision making and be sufficiently comfortable to be worn over long periods of time. Advancements in information technology and hardware manufacturing transformed former bulky and cable-bound AR headsets into ergonomic devices fulfilling strict requirements of ergonomic design [19]. However, as a result of the novelty of this technology and the lack of widespread application, data considering acceptance of surgeons is missing. We therefore performed a prospective clinical feasibility study in a real world-environment where orthopedic surgeons used a commercially available holographic headset (HoloLens I, Microsoft, Redmond, WA, USA) during surgery (Fig. 1). The surgeon’s experience of using the device during surgery was recorded using a standardized questionnaire.

## Methods

Orthopedic surgeons from a single Swiss university clinic where the subject of this prospective study. Surgeons were asked to wear a commercially available holographic AR headset (HoloLens, Microsoft, Redmond, WA, USA) during surgery after receiving an introduction explaining its functionality and time to test voice commands and hand gestures.

**Fig. 2:**
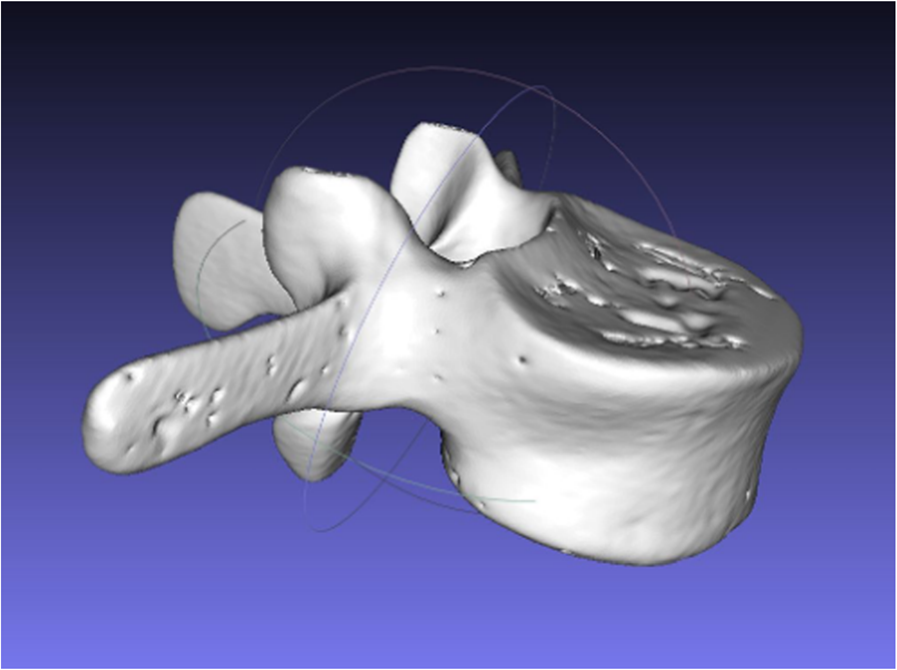
Three-dimensional model of a single vertebra generated from CT data

For each operation, a three-dimensional triangular surface model of the patients readily available CT data was generated using a commercial software (Siemens syngo.via Frontier 3D printing V 1.0.0, Siemens Healthineers, Erlangen, Germany or Materialise Mimics V 19.0, Leuven, Belgium) [20]. Only already available data was used, and no additional CT scans were performed for this study. The three-dimensional model (Fig. 2) was edited with the Unity software package (Version 5.5, Unity Technologies, San Francisco, CA, USA). An application that permitted interactive rotation and translation of the three-dimension model by voice commands and contactless hand gestures was implemented using Microsoft Visual Studio 2015 (Microsoft, Redmond, WA, USA).

**Fig. 3:**
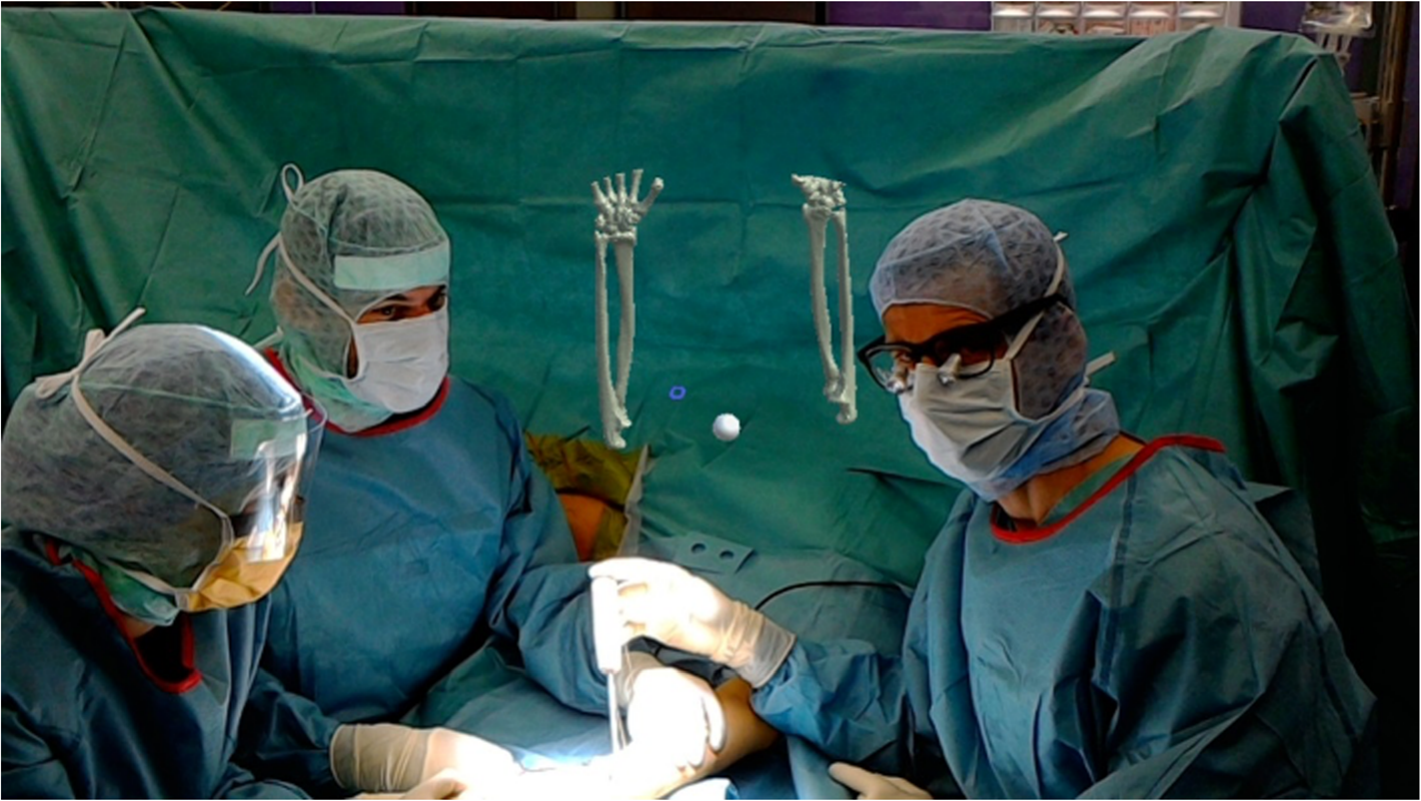
Surgeon wearing an augmented reality headset during surgery for shoulder arthroplasty using three-dimensional anatomic data supplied by the augmented reality headset

**Fig. 4:**
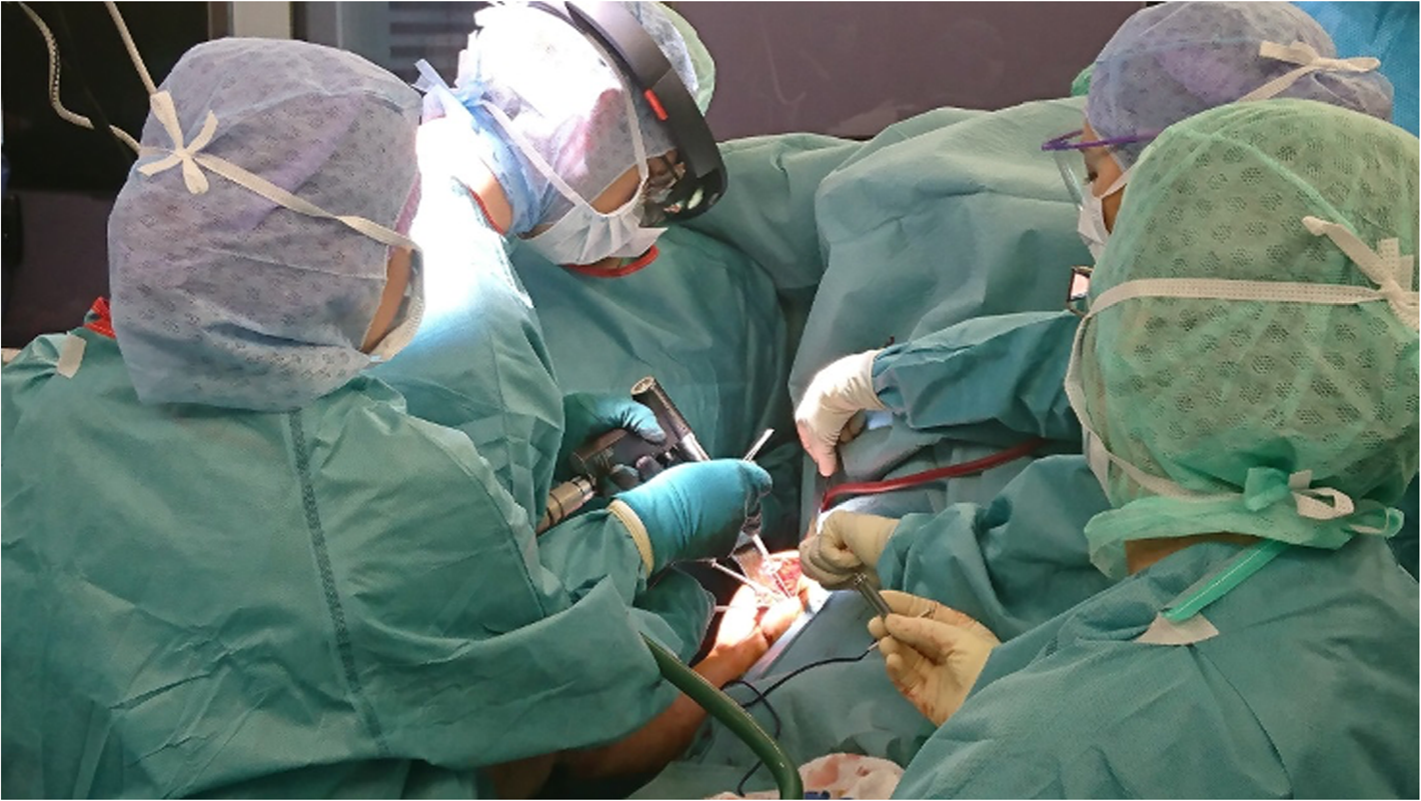
View from the surgeon’s perspective. Both forearms are displayed as 3D virtual objects during corrective forearm osteotomy

During the operation, the three-dimensional model of the patients CT-data was displayed holographically in the surgeon’s field of view in addition to routinely mounted radiological imaging on conventional screens. The displayed surface model could be moved, rotated, scaled and placed using contactless hand gestures and voice commands (Fig. 3). The timing and duration of the intraoperative use of the head mounted device was at the surgeon’s discretion (Fig. 4).

The device was evaluated using a standardized 58-item questionnaire administered as an electronic survey using the REDCap data capture tool [21]. The survey used a 100-point scale with anchor statements (1: not useful at all, to 100: very useful) to grade usability of the device, intraoperative implementation, benefit during the procedure, future potential of AR technology in general and considering orthopedic subspecialties as well as evaluation of the device itself. Further, demographic data considering the participating surgeons were recorded. Surgeons where also asked about the future potential of AR technology. A translated English version of this survey originally supplied to the participants in German is available as a supplementary file.

### Statistical analysis

Descriptive analysis was performed using SPSS (IBM Corp. Released 2017. IBM SPSS Statistics for Windows, Version 25.0. Armonk, NY: IBM Corp.). Continuous variables are reported as mean ± standard deviation (SD).

## Results

Thirteen orthopedic surgeons from different subspecialties (all male, mean age 40 ± 7 years) participated in this study. Four surgeons performed > 500 cases per year, 3 surgeons 251–500 and 6 surgeons 51–250 surgeries per year. Six surgeons were in the position of chief of service and 7 surgeons were attending physicians or fellows. In total, 25 surgeries (10 spine, 4 shoulder, 5 knee, 1 hand and 5 ft) were included in this study.

**Fig. 5:**
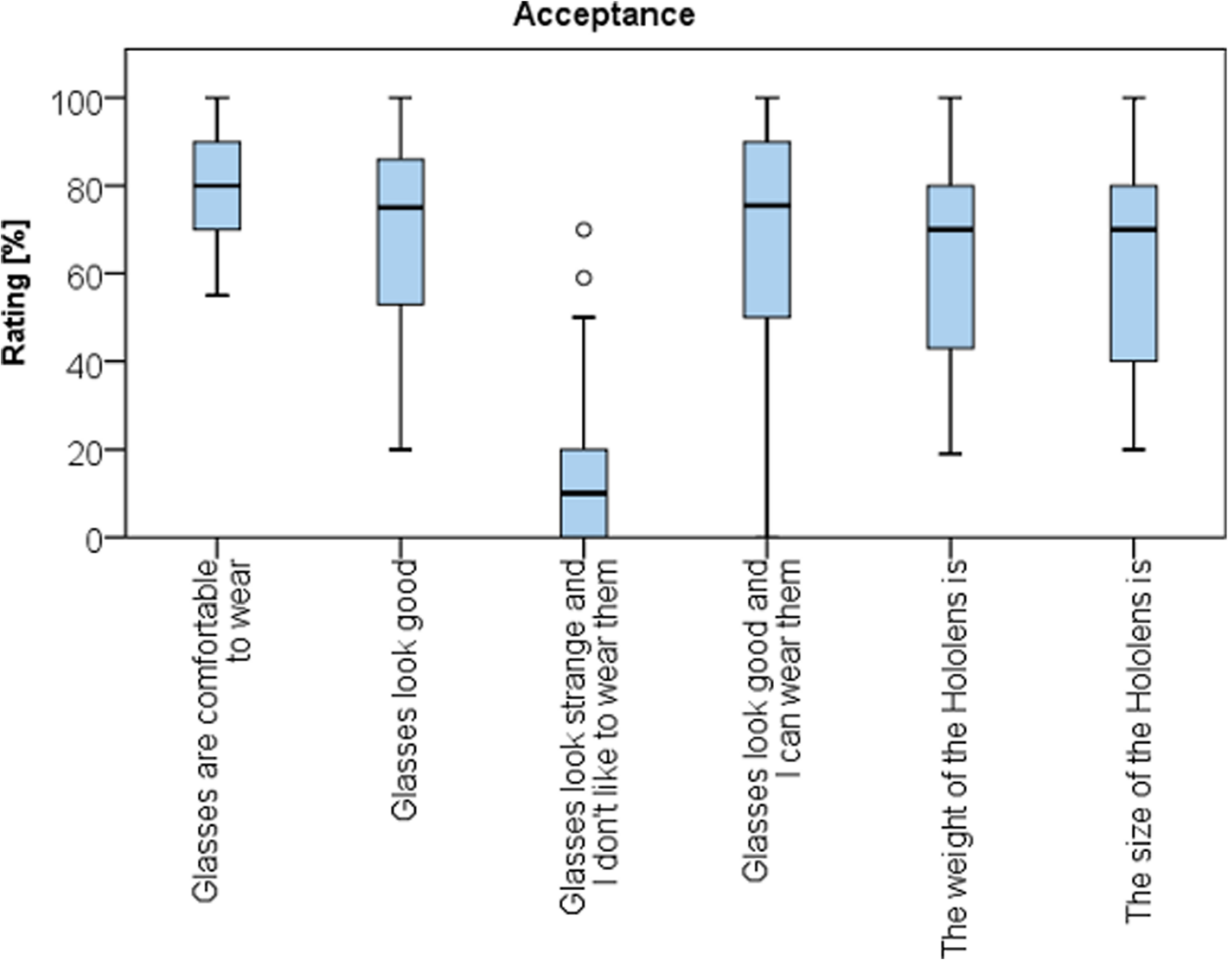
Boxplot showing general acceptance of the AR headset

The general acceptance of the AR device was good (Fig. 5). Wearing the AR device was rated as fairly comfortable (79 ± 13 points). Weight and size of the device were rated with 64 ± 23 points.

**Fig. 6:**
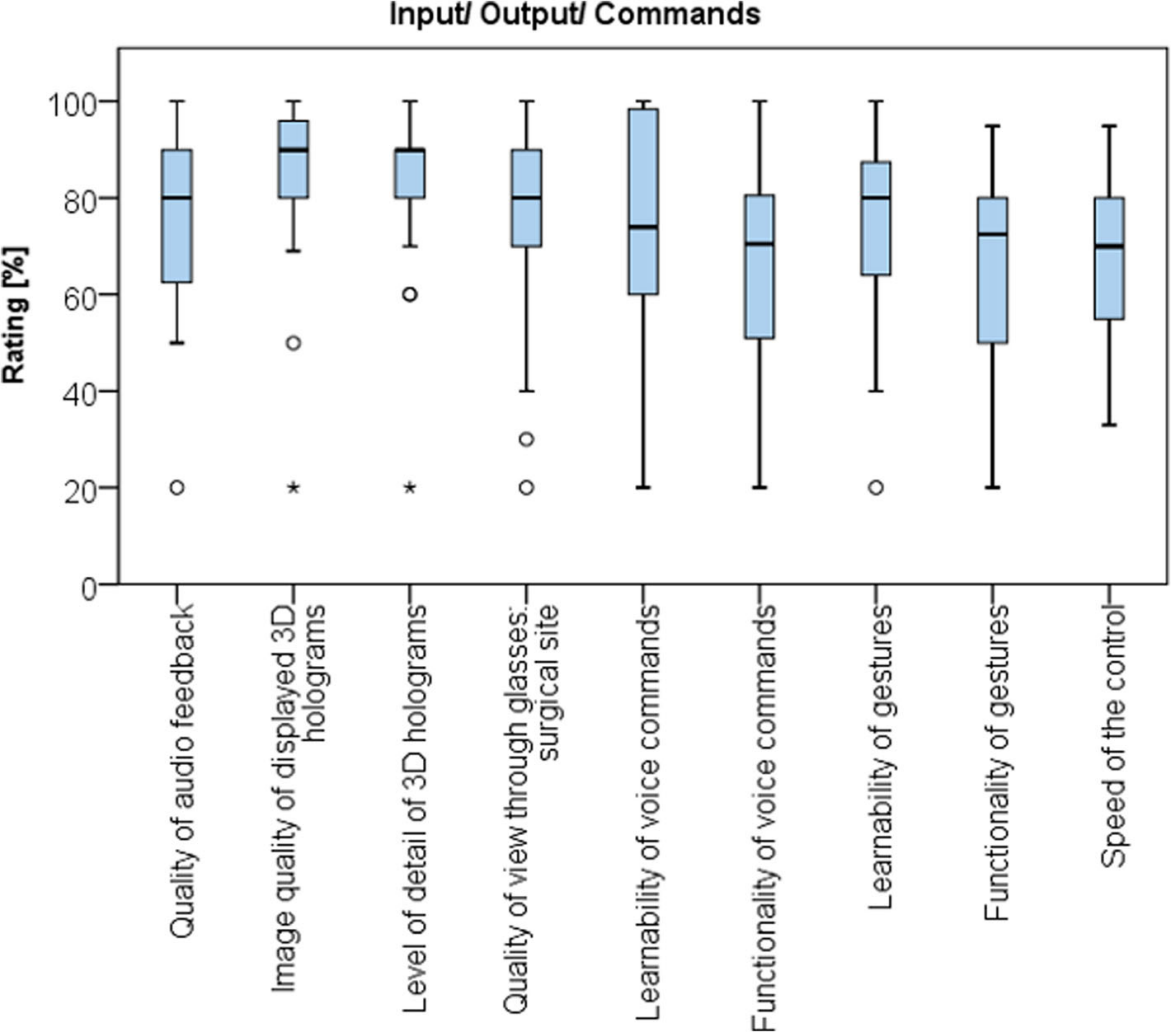
Boxplot showing the usability of the AR headset within the operating room

Considering usability (Fig. 6), surgeons found the device to produce good image quality (85 ± 17 points) and a good accuracy of virtual objects (84 ± 19 point). In contrast, functionality of voice commands (68 ± 20 points) and gestures (66 ± 20 points) provided less favorable results. Learnability of the voice commands (74 ± 23 points) and gestures (73 ± 20 points) were well rated. Voice control most frequently failed due to a lack of understanding of commands by the device (8/25) or because of noise in the surroundings (8/25). Overall satisfaction with the speed of the application was rated 69 ± 18 points.

**Fig. 7:**
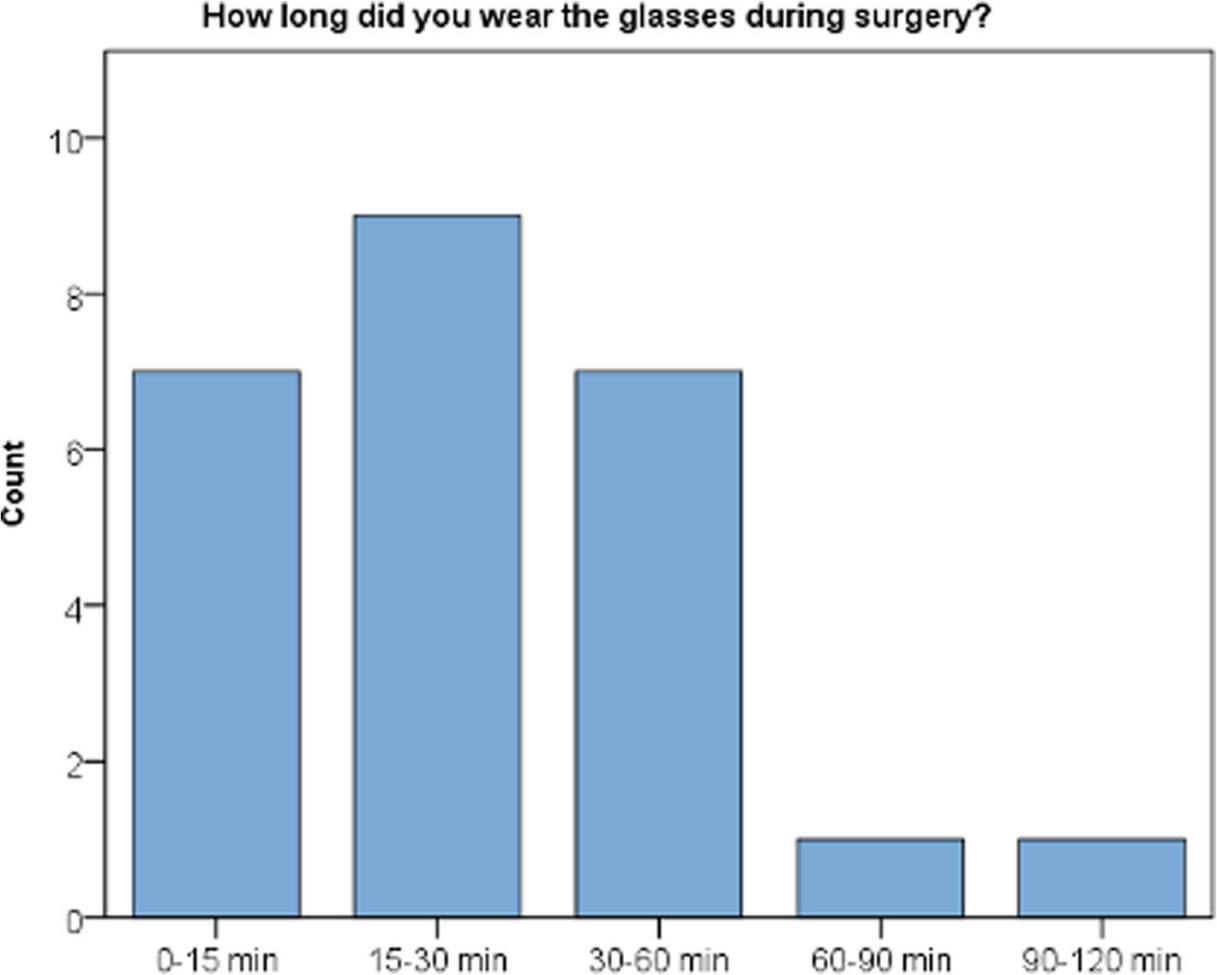
Bar chart showing wearing time of augmented reality headset during surgery

Most surgeons used the AR device for less than 60 min (23/25 surgeries) and no surgeon used the device for the entire surgery. All but three surgeons used the AR device only for preoperatively defined surgical steps (Fig. 7).

**Fig. 8:**
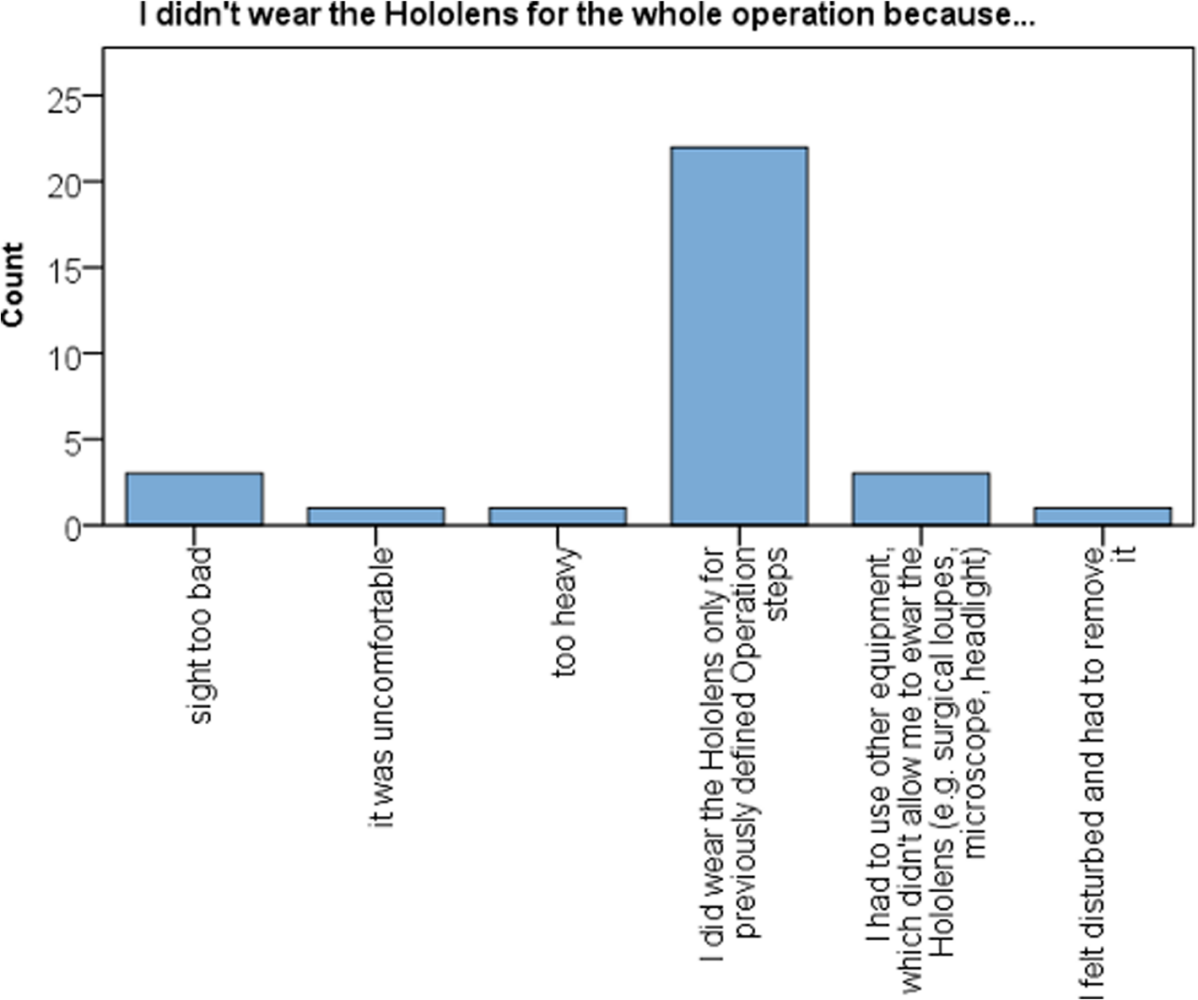
Bar chart showing reason for not wearing the AR headset during hole procedure

The most frequent reasons for early termination of use were impaired sight caused by the device (3/25) and surgical steps requiring other instruments (3/25) (Fig. 8). However, most surgeons estimated that they could wear the AR device for more than 60 min.

**Fig. 9:**
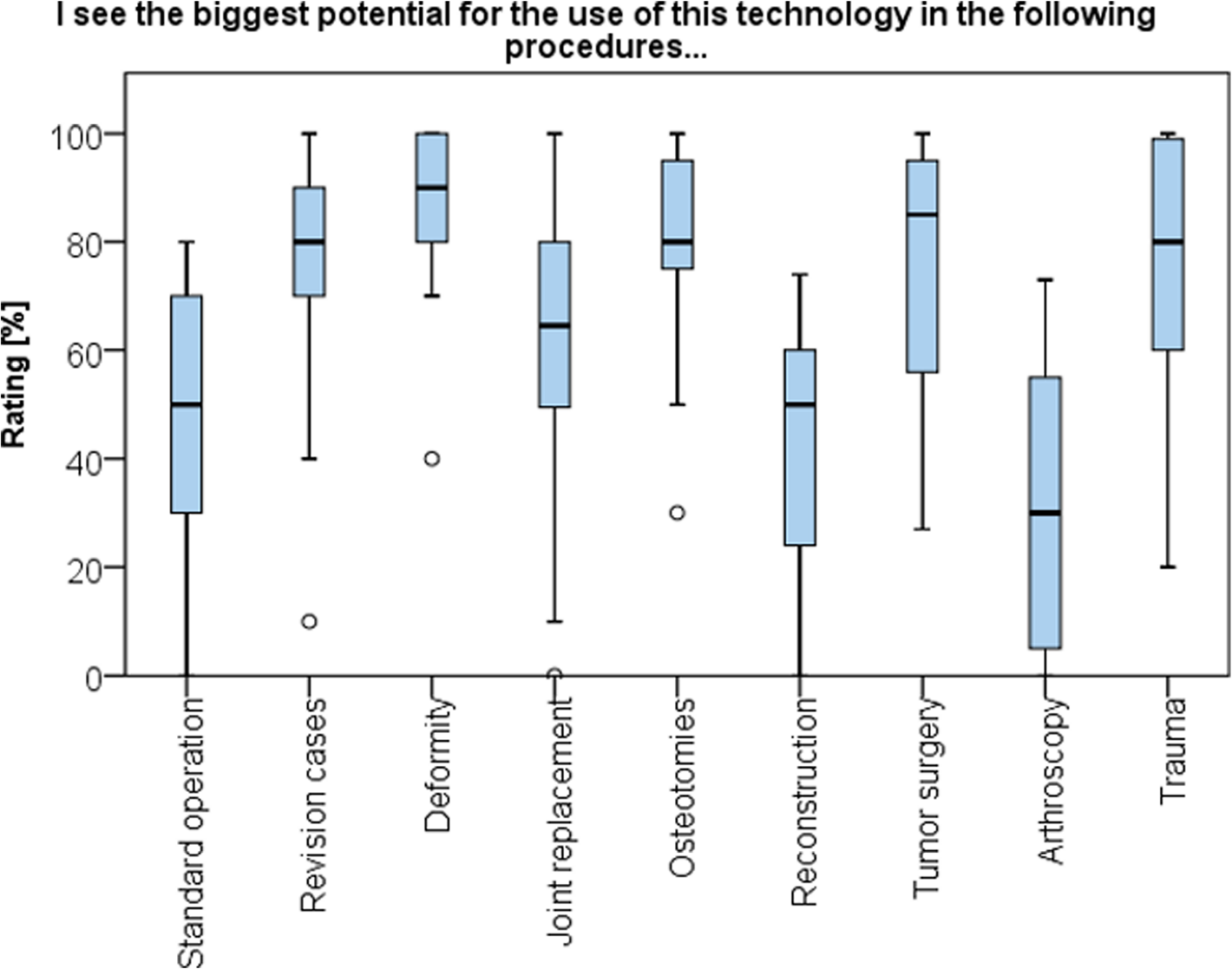
Boxplot showing the future potential for AR technology referring to surgical techniques in orthopedic surgery

The greatest potential in the use of the AR device was found for surgical correction of deformities (87 ± 15 points), osteotomies (82 ± 17 points), revision surgery (77 ± 22 points), and tumor surgery (77 ± 21 points), whereas lowest potential was found for arthroscopic (32 ± 25 points) and reconstructive surgery (41 ± 22 points) (Fig. 9).

Potential for future use within the orthopedic subspecialties was highest estimated for application in spine (86 ± 13 points) and pelvic surgery (88 ± 13 points).

**Fig. 10:**
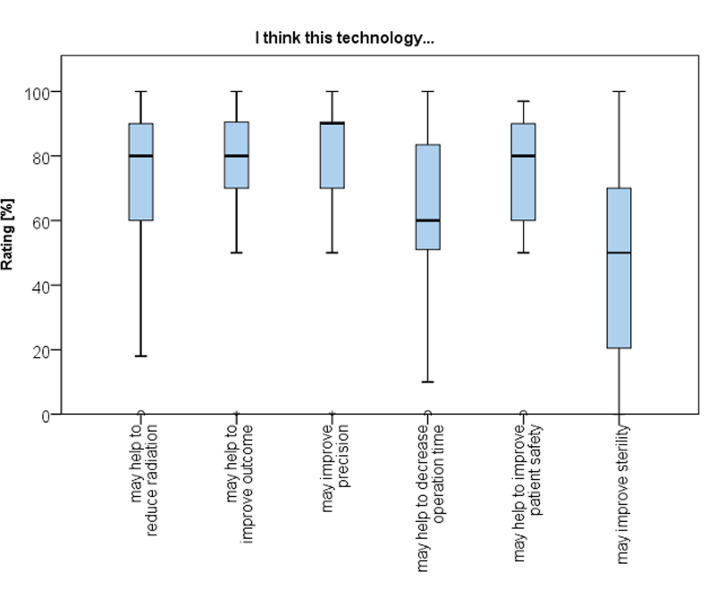
Boxpot showing expectations in augmented reality for surgical applications after usage

The greatest benefits of AR technology were expected to be increased intraoperative accuracy (78 ± 23 points), improved surgical outcome (77 ± 22 points) and reduction of exposure to radiation (72 ± 26 points) (Fig. 10).

Overall, surgeons were satisfied with the application of this novel technology (78 ± 20 points) and future access to it was demanded (75 ± 22 points).

## Discussion

This prospective clinical study offers a proof of concept of the clinical feasibility of a wearable augmented reality device for using three-dimensional virtual object reconstructions of individual patients’ anatomy as an intraoperative aid during orthopedic surgical procedures. Thirteen surgeons from different orthopedic subspecialties used an AR headset as an additional display providing complementary three-dimensional, patient specific anatomic information during 25 surgical procedures. The surgeons experience with the device was recorded using a standardized questionnaire.

Augmented reality is rapidly developing technology that can display a vast variety of information within the field of view of the user using a compact device with minimum infrastructure. Spatial mapping and recognition of the physical environment is a crucial difference between augmented reality and other navigations systems using conventional display methods. Projecting the required information directly into the line-of sight of the surgeon is considered the natural progression of these well-established methods mitigating the errors associated with attention shift by directly projecting the navigation guidance onto the surgical field [22, 23]. Incekara et al. evaluated the same AR headset for pre-operative planning of neurosurgical tumor resection in 25 patients. The authors reported that surgeons did benefit in terms of maintaining attention and focus on the patient, improved ergonomics and improved understanding of tumor-brain/ skull relationship due to direct three-dimensional holographic representation. In contrast to current neuronavigational systems, the HoloLens AR head mounted device subjectively improved ergonomics during surgical planning [24]. While projecting information directly into field of view of the surgeon is considered to mitigate errors associated with attention shift, the fixed focal distance of two meters of the virtual objects projected by the HoloLens could induce perceptual conflicts as focal rivalry and vergence-accommodation mismatch [23, 25]. However, because virtual objects projected in the here tested scenario were not directly overlayed with the patient anatomy, focal rivalry did not play an important role in this analysis.

The here used iteration of the HoloLens offers a rather narrow diagonal angle of view of 34° for projection of virtual objects. However, for most surgical tasks requiring high precision, a narrow field of view is sufficient and this study, surgeons were generally satisfied with the quality of the displayed three-dimensional images.

The general acceptance of the device was fairly good. Considering comfort, the mostly frontloaded weight of 579 g and the size of this first iteration of the HoloLens was considered as a shortcoming. During 6 of 25 cases, the device was worn for more than 30 min, and in another 7 of 25 cases, for 15–30 min. However, despite its weight and size, most surgeons participating in this study estimated that the device could be worn for more than an hour when considering comfort. Other authors investigating the usability of the HoloLens during neurosurgical procedures graded the comfort more favorable [24, 26]. This might be attributed to the fact that surgical subspecialties more accustomed to the use of optical aids in their field of view including magnification glasses might also be less disturbed be head mounted AR devices. In this study, the small number of surgeons wearing the device for more than 1 h (*n* = 2) might also be explained by the fact that participants were only supplied with supplementary information required for essential steps of the surgery. Hence, the threshold for removing the device, in particular while performing the surgical approach and during wound closure was low.

The questionnaire also revealed technical limitations of the device, namely the voice command and gesture function. Using voice commands as a mean of interaction with the headset was less favorably graded. In most cases the recognition of voice commands was impaired by loud environment in the operation room. Other reports analyzing the usage of the HoloLens device for different surgical specialties reported more stable results using voice commands [26]. This difference in outcome might be attributed to the variety of noise emitting tools and instruments utilized in orthopedic surgery. However, further advances in technology will surely overcome these limitations and will lead to improved ergonomics and wearability.

Surgeons saw the biggest potential for this technology in complex surgical procedures including revision surgery, deformity correction, tumor surgery and trauma where typical anatomical landmarks are frequently not easily identifiable. However, a crucial step to provide reliable navigation in complex surgery is the registration process, the automated and precise overlay of virtual information with the real environment. Various methods of registration have been described including ultrasound-based techniques, reflective markers and non-invasive skin placed markers [22, 23, 27]. Automated registration of the patient’s anatomy is currently the focus of various projects in augmented reality research [16, 17, 28, 29]. Intraoperative manual surface digitization or machine learning based object detection offer the possibility to establish a correspondence between preoperatively acquired image data and intraoperative anatomy without further requiring intraoperative imaging [29, 30]. Further, the quality of alignment of the three-dimensional objects to the real world not only relies on the accuracy of the registration but also on the calibration of the optical see-through head-mounted display. Therefore, different calibrations methods to map the physical reality to the virtual scene have been proposed [31, 32]. However, in an experimental setting, navigated pedicle instrumentation with augmented reality-based head mounted devices achieved comparable results to commercially available navigation systems when using 3D image-based registration [33]. While this study is the first to describe ergonomic aspects and feasibility of using the HoloLens in a sterile setting in orthopedic surgery, its main limitation is the small number of procedures performed and the short time each surgeon spent with the device. Also, as a result of technical shortcomings not yet overcome, the full potential of AR as a mean to actually navigate the described procedures with pre-planned cutting planes or screw trajectories could not be demonstrated. However, a clinical study accounting for these deficits is planned by this study group. This study might also be prone to selection bias as recruiting was performed on a voluntary basis possibly including subjects more enthusiastic for novel technology.

## Conclusion

In summary, AR is a rapidly evolving technology with large potential in different surgical settings, offering the opportunity to provide a compact, low cost alternative requiring a minimum of infrastructure compared to conventional navigation systems. While surgeons where generally satisfied with image quality of the here tested head mounted AR device, some technical and ergonomic shortcomings were pointed out. This study serves demonstrates the feasibility of the use of an AR head mounted device in a real-world sterile setting in orthopedic surgery.

## Supplementary Information

**Additional file 1.**

## Data Availability

The datasets used and/or analysed during the current study are available from the corresponding author on reasonable request.

## Abbreviations

AR: Augmented reality
CT: Computed tomography
SPSS: Statistical Package for Social Science
