# Supplementary material for: Augmented reality in the operating room: a clinical feasibility study
Source: BMC Musculoskelet Disord. 2021 May 18;22:451. doi: 10.1186/s12891-021-04339-w (PMC8132365; doi:10.1186/s12891-021-04339-w)
Supplement: Supplementary file 1 — Additional file 1. [file 12891_2021_4339_MOESM1_ESM.pdf]

Original Survey from: Augmented Reality in the Operating Room: a Clinical Feasibility Study

Cyrill Dennler, MD[1], David E. Bauer MD[1], Anne-Gita Scheibler, MD[2], José Spirig, MD[1], Tobias Götschi, MSc[3], Philipp Fürnstahl, PhD[4], Mazda Farshad, MD, MPH[1]

[1] Spine Division, University Hospital Balgrist, University of Zürich, Switzerland

[2] Laboratory for biomechanics, University Hospital Balgrist, University of Zürich, Switzerland

[3] Computer Assisted Research and Development Group, University Hospital Balgrist, University of Zürich, Switzerland

Corresponding author:
Dr. David E. Bauer
Spine Division
University Hospital Balgrist
University of Zurich
Forchstrasse 340
8008 Zurich
Switzerland
Phone: +41 44 386 11 11
Fax: +41 44 386 11 09
E-mail: david.bauer@balgrist.ch

1) PID

____

2) OP date

____

3) Name of surgeon

____

4) Year of surgery

____

5) Assistant doctor function
senior physician /
team leader / team leader deputy / consultant

6) Operations performed per year
- 0-50
- 51-250
- 251-500
- >500

7) Team / joint shoulder
- Shoulder/Elbow
- Hand
- Spine
- Pelvis/Hip
- Knee
- Foot
- Tumour

8) Surgical steps the Hololens was used for
(e.g. orientation, drilling,
Screw placement, osteotomies etc.)

Input / Output / Control

9) Audio quality 0-100

10) Image quality of holograms 0-100

11) Detail of holograms 0-100

12) Image quality of the surgical field when looking through the glasses 0-100

13) Learnability of voice control 0-100

14) Voice control functionality 0-100

15) Voice control did not work because:
- the Hololens did not understand my voice

- the environmental noise from people was too high
- the ambient noise caused by equipment was too high (suction etc.)

16) Learnability of gesture control 0-100

17) Functionality of gesture control 0-100

18) Total speed of control 0-100

Acceptance

19) The glasses are comfortable to wear 0-100

20) The glasses look good 0-100

21) I think the glasses look strange and I
therefore do not want to wear them 0-100

22) I think the glasses look aesthetic and I
can therefore wear it well 0-100

23) The weight of the glasses is 0-100

24) The size of the glasses is 0-100

Wearing period

25) For how many minutes did you wear the device during the operation?
0-15 min
15-30 min
30-60 min
60-90 min
90-120 min
>120 min

26) The device was used during the whole operation
Yes/No

27) I only wore the Hololens for part of the surgery for the following reasons
- visibility was impaired
- too uncomfortable
- too heavy
- I have worn the Hololens only for certain pre-determined surgical steps
- I also had to use other devices that made it impossible to wear the hololens (e.g. microscope,
magnifying glasses)
- I felt disturbed and had to remove the hololens

28) I think it is possible to wear the Hololens continuously during an operation for the following period
- 0-30 min
- 30-60 min
- 60-90 min
- 90-120 min
- >120 min

Regarding the operation
I think this technology...

29) helps to reduce radiation exposure 0-100

30) helps to improve the surgical result 0-100

31) increases the precision 0-100

32) increases the speed of the operation 0-100

33) increases patient safety 0-100

34) improves sterility 0-100

18.10.2020 18:09 projectredcap.org

Future

I see the greatest potential for this technology to support the following operations

35) Standard operations 0-100

36) Revision cases 0-100

37) Deformities 0-100

38) Prosthetics 0-100

39) Osteotomies 0-100

40) Reconstructions (e.g. cruciate ligament) 0-100

41) Tumor surgery 0-100

42) Arthroscopic operations 0-100

43) Trauma 0-100

I see the greatest potential of this technology in operations on the following joints

44) Shoulder 0-100

45) Elbows 0-100

46) Hand 0-100

47) Spine 0-100

48) Basins 0-100

49) Hip 0-100

50) Knee 0-100

51) Foot 0-100

Overall verdict
52) How satisfied are you with this new technology? 0-100

53) I would like to use this technology again at any time
can fall back on 0-100
